# Consensus framework for developing a target product profile of real-time PCR in Chagas disease therapeutic monitoring

**DOI:** 10.1371/journal.pntd.0014452

**Published:** 2026-07-09

**Authors:** Alejandro G. Schijman, Colin Forsyth, Alba Abras, Igor C. Almeida, Julio Alonso-Padilla, Cristina Alonso-Vega, Cristina Ballart-Ferrer, Margarita Bisio, Constança Britto, Eric Dumonteil, María Flores-Chavez, Montserrat Gallego, Claudia P. Herrera-Bernal, Constanza Lopez-Albizu, Otacilio C. Moreira, Arturo Muñoz-Calderon, Lilian Pinto, Juan C. Ramirez, Ivan Scandale, María A. Shikanai-Yasuda, Sergio Sosa-Estani, María-Jesus Pinazo

**Affiliations:** 1 Laboratorio de Biologia Molecular de la Enfermedad de Chagas, Instituto de Investigaciones en Ingeniería Genética y Biología Molecular “Dr Héctor Torres” (INGEBI-CONICET), Buenos Aires, Argentina; 2 Chagas Program, Drugs for Neglected Diseases Initiative (DNDi) LATAM, Rio de Janeiro, Brazil; 3 Departamento de Biología, Universidad de Girona, Girona, Spain; 4 Department of Biological Sciences, University of Texas “El Paso”, El Paso, Texas, United States of America; 5 Instituto de Salud Global de Barcelona, Barcelona, Spain; 6 Centro de Investigación Biomédica en Red de Enfermedades Infecciosas (CIBERINFEC), Instituto de Salud Carlos III, Madrid, Spain; 7 INTERTRYP, Univ Montpellier, Cirad, IRD, Montpellier, France; 8 Departament de Biologia, Sanitat i Medi Ambient, Facultat de Farmàcia i Ciències de l’Alimentació, Universitat de Barcelona, Barcelona, Spain; 9 Instituto Nacional de Parasitología “Dr Mario Fatala Chaben” (ANLIS “Dr Carlos G. Malbrán”), Buenos Aires, Argentina; 10 Consejo Nacional de Investigaciones Científicas y Técnicas (CONICET), Buenos Aires, Argentina; 11 Lab. de Biologia Molecular e Doenças Endêmicas Instituto Oswaldo Cruz, FIOCRUZ, Rio de Janeiro, Brazil; 12 Department of Tropical Medicine and Infectious Disease, Celia Scott Weatherhead School of Public Health and Tropical Medicine, Tulane University; New Orleans, Louisiana, United States of America; 13 Instituto de Salud Carlos III, Madrid, Spain; 14 Laboratório de Virologia e Parasitologia Molecular, Instituto Oswaldo Cruz, Fundação Oswaldo Cruz, Rio de Janeiro, Brazil; 15 Fundación CEADES, Cochabamba, Bolivia; 16 Hospital de Niños Ricardo Gutierrez, Buenos Aires, Argentina; 17 Laboratorio de Parasitología Molecular, IMIPP (Instituto Multidisciplinario de Investigación en Patologías Pediátricas) CONICET – GCBA, Buenos Aires, Argentina; 18 Drug Discovery, Drugs for Neglected Diseases Initiative, Geneve, Switzerland; 19 Hospital das Clínicas da Faculdade de Medicina da Universidade de São Paulo (HCFMUSP), São Paulo, Brazil; University of Colorado Anschutz Medical Campus: University of Colorado - Anschutz Medical Campus, UNITED STATES OF AMERICA

## Abstract

Chagas disease remains a neglected global health problem with major diagnostic and therapeutic challenges. Progression from acute to chronic infection may lead to cardiac or digestive complications in approximately 20–40% of patients, depending on geographic region and duration of follow-up. However, reliable prognostic markers are still lacking, underscoring the importance of early diagnosis and timely treatment. For over 40 years, therapy has relied on benznidazole and nifurtimox, effective in acute but inconsistent in chronic disease, with frequent adverse effects undermining adherence. Current research is testing shorter regimens, lower doses, and novel compounds. Their evaluation is constrained by the lack of standardized biomarkers of treatment response. Serology requires decades to confirm treatment effect, whereas quantitative PCR (qPCR) enables earlier detection of therapeutic failure. However, heterogeneity of qPCR protocols hampers cross-trial comparisons and meta-analyses. To resolve these limitations, the Drugs for Neglected Diseases initiative convened experts to build consensus on qPCR application in clinical trials aiming to define a target product profile supporting drug development and regulatory approval.

## Introduction

Chagas disease (CD), caused by *Trypanosoma cruzi* (*T. cruzi*), remains a neglected disease with major unmet needs in diagnosis, prognosis, and treatment [1]. Although historically confined to the Americas, population mobility has turned CD into a global health concern. Infection typically progresses from an acute phase with variable or mild symptoms to a chronic stage in which about 70% of the infected individuals remain asymptomatic. However, 20–40% of those chronically infected, depending on geographic region and duration of follow-up will develop cardiac and/or digestive complications years or decades later. At present, no validated biomarkers predict disease progression in chronic CD [2], making early diagnosis and timely treatment essential to prevent irreversible organ damage.

For more than four decades, treatment has relied almost exclusively on the nitroimidazoles nifurtimox (NFX) and benznidazole (BNZ). These drugs are effective during the acute phase, but their efficacy in the chronic phase is variable and influenced by parasite lineage and host factors. Moreover, treatment benefit has not been demonstrated in those patients with established advanced cardiomyopathy [3]. Despite these limitations, treatment of infected women before pregnancy significantly reduces congenital transmission. Current regimens require 60 days of therapy and frequently cause adverse events that limit adherence. As a result, clinical research is exploring shorter or lower-dose regimens, drug re-purposing, and novel compounds or combinations. Several ongoing trials are evaluating whether modified dosing of BNZ or NFX can maintain efficacy while reducing toxicity [4–7].

The evaluation of new therapies faces the same fundamental obstacle encountered with BNZ and NFX: the lack of robust biomarkers of therapeutic response or cure. Parasitological methods remain the gold standard for detecting treatment failure, while serological reversion of anti-*T. cruzi* IgG is widely accepted as evidence of cure. However, seroconversion may take decades in chronic CD, making it impractical for clinical trials and the clinical routine. Consequently, no consensus exists on how to define treatment efficacy in chronic infection [1]. Molecular methods, particularly real-time quantitative PCR (qPCR), can detect early treatment failure but do not constitute definitive tests of cure [8–11].

Quantitative PCR has been extensively used to quantify reductions in parasite load following treatment, yet relapse after apparent clearance is common. In the STOP-Chagas and Chagasazol trials, 90% of patients treated with posaconazole became PCR-positive during follow-up [12,13]. Similarly, the E1224 trial showed only transient parasite clearance with low-dose regimens, while sustained responses were achieved in a minority of patients receiving high-dose therapy [14]. By contrast, BNZ achieved sustained parasite clearance in most patients, although long-term failure rates remained high, reaching 39.6% in the study performed by Galvão and coworkers [15], 46% in the BENEFIT trial [3] and up to 88.3% after 12 months in Brazilian participants of the MULTIBENZ study [7].

Despite being the most accurate available tool to monitor parasitological response in Phase II and selected Phase III trials, qPCR has important limitations. Follow-up periods are usually limited to 12 months, which is insufficient to establish cure, and methodological heterogeneity across studies compromises comparability. Critical parameters—including blood volume, sample preservation, DNA extraction, and assay design—strongly influence sensitivity [11,16].

Several methodological improvements have enhanced qPCR performance, including multiplex assays, serial blood sampling, and automated DNA extraction [17–22].

External quality assessment programs have supported inter-laboratory proficiency testing [23] while commercial qPCR kits—approved in some endemic countries—may improve reproducibility when strict standard operating procedures (SOPs) are followed [16,24,25].

Given the critical role of qPCR in drug development and the challenges posed by its variability, the Drugs for Neglected Diseases initiative (DNDi) convened an expert panel in Chicago in October 2023 to establish consensus recommendations on qPCR use in CD clinical trials. This effort aimed to define performance criteria and lay the groundwork for a Target Product Profile (TPP) to guide assay development, trial design, and regulatory evaluation.

## Methods

To address the lack of standardization in qPCR-based monitoring of CD treatment, DNDi convened a multidisciplinary panel of experts from healthcare, academic, and research institutions across the Americas and Europe (Table 1). Participants were selected based on their recognized leadership in CD research and clinical management.

**Table 1:** List of experts participating in the Consensus Meeting for developing a Target Product Profile of qPCR in clinical trials.

Participant	Affiliation	Country
Abras, Alba	Universitat de Girona, Girona	Spain
Almeida, Igor C	University of Texas El Paso	United States of America
Alonso-Padilla, Julio	Instituto Salud Global Barcelona	Spain
Alonso-Vega, Cristina	Instituto Salud Global Barcelona	Bolivia
Altcheh, Jaime	Servicio Parasitología- Chagas	Argentina
	Hospital de Niños Ricardo Gutierrez	
	IMIPP, Instituto Multidisciplinario de Investigación en Patologías Pediátricas	
	CONICET - Gobierno de la Ciudad de Buenos Aires	
Ballart Ferrer, Cristina	Universitat de Barcelona	Spain
Barreira, Fabiana	Drugs for Neglected Disease Initiative	Brazil
Bisio, Margarita	Instituto Nacional de Parasitología Mario Fatala Chaben (ANLIS Carlos Malbrán)	Argentina
Britto, Constança	Instituto Oswaldo Cruz	Brazil
Buekens, Pierre M	University of Tulane	United States of America
Cordero, Elisa	Instituto de Biomedicina de Sevilla (IBiS)	Spain
Cucunubá Pérez, Zulma	Instituto Nacional de Salud	Colombia
Dumonteil, Eric O.	University of Tulane	United States of America
Flores Chavez Maria	Instituto de Salud Carlos III	Spain
Forsyth, Colin	Drugs for Neglected Disease Initiative	United States of America
Fraisse, Laurent	Drugs for Neglected Disease Initiative	France
Gallego Cullere, Montserrat	Universitat de Barcelona	Spain
Herrera, Claudia P	University of Tulane	United States of America
Hochberg, Natasha	Novartis	United States of America
Lopez Albizu, Constanza	Instituto Nacional de Parasitología Dr. Mario Fatala Chaben (ANLIS Carlos Malbrán)	Argentina
Meymandy, Sheba	University of California	United States of America
Molina, Israel	Hospitall Vall de Ebron	Spain
Moreira, Otacilio	Instituto Oswaldo Cruz	Brazil
Muñoz-Calderon, Arturo	LabMECh, INGEBI-CONICET	Argentina
Pinazo, María-Jesus	Drugs for Neglected Disease Initiative	Bolivia
Pinto, Lilian	Fundación CEADES	Bolivia
Ramirez, Juan Carlos	Hospital de Niños Ricardo Gutierrez	Argentina
	IMIPP-CONICET-GCBA	
Scandale, Ivan	Drugs for Neglected Disease Initiative	Switzerland
Schijman, Alejandro Gabriel	LabMECh, INGEBI-CONICET	Argentina
Shikanai Yasuda, Maria A.	Universidade de São Paulo	Brazil
Sosa-Estani, Sergio	Drugs for Neglected Disease Initiative	Brazil
Rao, Srinivasa	Novartis	United States of America
Villar, Juan Carlos	Fundación Cardioinfantil La Cardio	Colombia

The process began with a comprehensive literature review and technical assessment of qPCR methodologies, which revealed substantial variability in protocols and result interpretation. These findings informed iterative online discussions and two in-person meetings: an international consensus workshop in Chicago (October 19, 2023) and the DNDi Chagas Platform plenary in Buenos Aires (May 9, 2024).

Discussions focused on defining acceptable and optimal conditions for qPCR use in treatment monitoring within clinical trials. Where empirical evidence was limited, expert consensus was used to establish recommendations. The resulting target characteristics were framed according to WHO guidance on TPPs [26] and are summarized in Tables 2–6.

**Table 2:** Key qPCR attributes for defining a Target Product Profile in clinical trials of CD.

Attribute	Rationale and notes
**1. Intended use and target population**	To support standardized outcome assessment in trials and ensure applicability across diverse study populations. It can be implemented in the follow-up of neonates infected with *T. cruzi*, pediatric acute and chronic cases, adult acute and chronic cases, and people with any immunosuppressive condition (HIV infection/AIDS, solid organ, and/or hematopoietic stem cell (HSCT) transplantation, autoimmune disease, and people on immunosuppressive therapy).
**2. Trial duration and qPCR testing timeline points**	The trial duration should be sufficient to demonstrate treatment failure and, ideally, long enough to assess sustained efficacy. The testing schedule should include, at minimum, baseline assessment before treatment initiation and periodic post-treatment evaluations through the end of the trial, enabling detection of treatment failure based on at least one qPCR-positive result after treatment completion.
**3. Laboratory requirements and Target users**	To allow decentralized processing in multi-center trials, it is crucial to address the competencies of laboratory technicians, biomedical professionals, and biochemists, by ensuring training and performing the quality control needed for conducting qPCR. Requirements for setting up a reliable real-time PCR, including laboratory infrastructure and quality certifications, equipment, and reagent storage conditions. Adaptability to various research settings.
**4. Sample type and processing**	Selection of appropriate samples for qPCR, adapted to field conditions and with simple logistics. Regarding processing, the following should be considered: - Use of anticoagulants and stabilizing agents: recommendations for maintaining sample integrity. - Sample volume: the required volume of biological material. - Sample conservation and transportation: guidelines for maintaining sample quality during storage and transport. - Number of samples needed for monitoring, which should include baseline sampling (initial sample collection before treatment) and samples collected at specific intervals during and after treatment for follow-up. - Sample replicates: the number of blood sample aliquots to be processed for DNA extraction at each patient visit. - Sample processing: DNA extraction procedures for downstream qPCR testing.
**5. Target analyte**	The most appropriate *T. cruzi* DNA sequence in terms of sensitivity (multicopy and highly conserved among all *T. cruzi* discrete typing units) and specificity (present in *T. cruzi* but absent in *Leishmania* spp. and other trypanosomatid species that could generate a false-positive result with qPCR).
**6. Cycle threshold and assay variability**	The optimal upper Ct value to define qPCR positivity should be validated during assay implementation for each qPCR assay and thermocycler, using appropriate positive and negative controls.
**7. Analytical sensitivity and specificity**	Analytical sensitivity aims to ensure detection of low levels of parasitic loads after treatment. It is related to the LOD_95_ of the procedure: the ability of the qPCR assay to detect even the lowest concentration of the target nucleic acid in a sample. Analytical specificity aims to reduce false positives during follow-up. It is also related to test exclusivity: ensuring the assay does not cross-react with nontarget organisms potentially present in the sample tested.
**8. Parasite load quantification**	Quantitative PCR allows dynamic monitoring of parasitemia, but requires the establishment of standardized calibration curves and reporting units. It is important to determine the limit of quantification for the technique, i.e., the minimum DNA quantity that can be reliably quantified with minimal accuracy (LOQ).
**9. Internal and external quality controls**	Definition of the types of internal and external quality controls required to ensure the accuracy and reliability of qPCR results and to provide a harmonized methodology across multiple PCR laboratories performing the analysis in multi-center trials.
**10. Clinical sensitivity**	Adequate clinical sensitivity is essential to accurately identify cases of treatment failure. Clinical sensitivity is influenced by the number of blood sample replicates, number of DNA extraction replicates per each blood sample replicate and number of qPCR replicates per each DNA lysate.
**11. Clinical specificity**	Avoids misclassification of treatment failure due to false-positive results.
**12. qPCR data interpretation**	Enables consistent analysis across study centers.
**13. Time to result**	Timely reporting enables protocol adherence and effective patient management.
**14. Genotyping of Discrete Typing Units**	Complementary information to qPCR findings.

**Table 3:** Clinical trial duration and qPCR testing timeline points.

Category	Acceptable conditions	Optimal conditions
**Duration of trial**	**12 months**	**36 months**
**Screening/pretreatment** **(M0)**	One visit	One visit
**End of treatment (M1)**	Yes	Yes (after 5 half-life times of the used drug)
**Post-treatment**	2 points between M1 and M6 post-treatment: M2 and M4 2 points between M6 and M12 post-treatment: M8 and M12	2 points between M1 and M6: M2 and M4 2 points between M6 and M12: M8 and M12 and annually until the end of follow-up.

**Table 4:** Laboratory requirements and target users.

Category	Acceptable condition	Optimal condition
**Laboratory infrastructure and qualification**	High-complexity hospital or reference laboratory. Quality control certified laboratories.	Low-complexity hospital (2nd level). Quality control certified laboratories.
**Operator qualification**	Biochemist or trained technician.	Well-trained laboratory technician with expertise in gene amplification assays
**Amplification assays**	Validated *in-house* qPCR assays or commercial kits based on TaqMan approaches, with the use of internal amplification controls.	Validated commercial kits based on duplex TaqMan approaches with internal amplification control.
**Additional Comments**	Instruments need to be widely available with a low total cost of consumables and instruments. Health authority requirements may vary by country. Instrumentation may need flexibility depending on the context of use (e.g., future commercial/community settings).	Analytical parameters of PCR, specific to each kit, must be validated on the device to be used.

**Table 5:** Sample type and processing.

Category	Acceptable Condition	Optimal Condition
**Sample type**	Blood tube with Guanidine Hydrochloride 6M- EDTA 0.2 M (GEB), pH 8.0 buffer (for manual DNA extraction), or EDTA alone (for automatic DNA extraction).	EDTA and/or filter paper for automatic DNA extraction.
**Sample conservation and transport**	Need to assess the maximum period for maintaining sample stability. GEB at room temperature for 1 month or EDTA at −20 °C. Filter paper at room temperature or refrigerated for at least 6 months.	GEB at room temperature or EDTA at −20 °C for at least 1 year. Filter paper at room temperature for at least 1 year
**Sample volume**	Minimum sample volumes: 5 mL for adults, 1 mL for children, and 125 µL for dried blood spots (DBS). Whenever possible, the collection of additional sample volume is recommended to ensure availability for backup testing.	1 mL for adult patients, 0.5 mL for children, 125 µL for filter paper (DBS).
**Sample number**	Screening/pretreatment: one sample, and if negative, a second blood extraction. Post-treatment: one sample per timeline point.	Screening/pretreatment: one sample. Post-treatment: one sample per timeline point.
**DNA extraction replicates**	One DNA extraction in acute cases. Two or three DNA extractions per sample/visit in chronic CD patients.	A single DNA extraction for any setting.
**qPCR replicates**	Duplicate or triplicate PCR measurement per each DNA extract	Each DNA extract should be tested at least in duplicate by PCR, and the mean ± SD parasitic load value of both replicates should be reported.

**Table 6:** Analytical performance of qPCR assays and quality assessment.

Parameter	Acceptable conditions	Optimal conditions
**Inclusivity**	**Detects all *T. cruzi* strains and Discrete Typing Units (DTUs)**	
**Analytical sensitivity**	**Limit of Detection (LoD**_**95**_ **): 1 parasite genome equivalent/mL blood**	**LoD**_**95**_ **: 0.1–0.5 parasite genome equivalents/mL blood**
	**Higher sensitivity than blood microscopy tests (Micromethod, Strout)**	
**Analytical specificity**	**No detection of Leishmania sp., low level detection of T. rangeli**	**No detection of *Leishmania* sp or *T. rangeli***
**Quantification of parasitic loads**	**Not mandatory. If the assay includes a quantification curve, parasitic loads can be expressed in parasite equivalents or copies of molecular target per unit of sample volume**	**Report of parasitic loads, expressed in copies of the molecular target per unit of sample volume. Standard curve generated using a synthetic DNA with sequence of molecular target**
**Internationally accepted internal reference Standards**	**Each qPCR assay run should include strong and weak positive controls, a nontemplate control, an internal amplification control, and a negative DNA extraction control to monitor intra- and inter-laboratory operational variability, instrument performance differences, and run-to-run variation.**	
**Endogenous patient control genes:**	**Include an endogenous human DNA target to assess sample quality, storage/transport conditions, DNA extraction efficiency, patient-specific factors, and PCR inhibition. An exogenous IAC may alternatively be used to monitor extraction efficiency and PCR inhibition.**	**Internal amplification controls in multiplex format**
**External quality assessment**	**Participate in semiannual proficiency testing and use contrived sample panels, consisting of sample matrices spiked with known parasite loads, to monitor the end-to-end process from DNA extraction to qPCR readout.**	**Participate in semiannual proficiency testing programs and perform blinded re-testing of a random selection of clinical trial samples.**

## Results

### Intended use and target population

Quantitative PCR was defined as a surrogate marker of parasitological response applicable across a broad range of patients, including infants with congenital CD, pediatric and adult patients with acute or chronic infection, and immunosuppressed individuals. However, while qPCR has demonstrated reliability for detecting treatment failure, its role as a surrogate for sustained parasitological response requires further validation through correlation with clinical and serological outcomes in longer-term studies (Table 2).

### Trial duration and qPCR timelines

Consensus was reached on acceptable and optimal sampling points (screening, pre-treatment, end-of-treatment, and follow-up) and trial duration (Table 3).

Defining treatment failure on the basis of qPCR positivity supports the use of a 12-month post-treatment follow-up as a pragmatic interim window, given the timing of most documented relapses. Available evidence indicates that most qPCR-detectable treatment failures occur within the first 3–6 months after treatment completion, with nearly all molecular relapses becoming evident during this early period [6,7,10,14,27].

However, qPCR negativity in peripheral blood should not be interpreted as evidence of sterilizing cure or complete parasite clearance. Rather, qPCR is best framed as a tool primarily validated for the early detection of treatment failure, defined as the reappearance of detectable parasite DNA in blood. Its negative predictive value for persistent tissue-level infection, and therefore for sustained parasitological response, remains uncertain.

On this basis, efficacy assessment could be organized in two complementary stages: first, a 12-month interim endpoint aimed at detecting early treatment failure; and second, a 24–36-month confirmatory endpoint to evaluate sustained parasitological clearance. This stratified approach is consistent with the framework summarized in Table 3.

Extending follow-up beyond 12 months would strengthen the validation of qPCR as a surrogate marker of sustained treatment response [5]. Nevertheless, longer follow-up also introduces important feasibility challenges, including increased logistical complexity, higher costs, and greater risk of patient attrition. Loss to follow-up may bias relapse estimates, particularly if it is substantial or differs between treatment groups.

### Laboratory requirements

Acceptable laboratory settings include GLP-compliant facilities with physical separation of pre- and post-PCR areas, ideally with controlled pressure workflows. Although qPCR can be performed by trained technicians, the most appropriate standard is demonstrated competence validated through: (1) participation in harmonization studies that demonstrate proficiency in performing the specific qPCR protocols to be used in the trial; (2) successful performance in proficiency testing programs (internal or external quality assessment schemes) with documented acceptable results; (3) Good Laboratory Practice (GLP) training or equivalent quality management system training appropriate to the regulatory context of the trial; and (4) documented validation experience with the specific qPCR assay platform and protocols being implemented. This competency-based approach: (i) ensures quality through objective performance metrics rather than relying on proxy measures like years of experience; (ii) accommodates diverse settings including sites in endemic countries where formal certification programs may differ; (iii) focuses on demonstrated ability to perform the specific techniques required for the trial; and (iv) aligns with regulatory expectations for operator qualification in clinical trials.

Participating laboratories should implement quality systems, SOPs, and international certification where possible (Table 4).

### Sample type and processing

Consensus recommendations were established for blood collection, anticoagulants, stabilizers, sample volume, transport, processing, and replicate sampling (Table 5).

Serial sampling and replicate testing can enhance detection sensitivity, but they should be considered as trade-offs within a TPP, given their implications for sample volume, laboratory workload, cost, and turnaround time. Because sampling frequency and assay sensitivity are key determinants of the ability to detect intermittent parasitemia in chronic CD, trial protocols should explicitly define and justify both parameters.

### Molecular target analyte

Nuclear satellite DNA (satDNA) remains the most widely used target. Single extraction/qPCR is acceptable for acute cases, whereas triplicate testing is recommended for chronic infection. Minicircle DNA (kDNA) assays show promise for higher sensitivity but require further validation.

### Ct thresholds and assay variability

Variation among thermocyclers and assays complicates definition of the upper Ct cutoff. Laboratories must validate assay-specific Ct thresholds using appropriate controls, particularly for post-treatment samples.

### Analytical performance

Consensus criteria for analytical sensitivity, specificity, and parasite load quantification are summarized in Table 6.

A conservative, achievable limit of detection (LoD_95_) target of 1 parasite-equivalent per mL (par. eq./mL) was established to balance analytical sensitivity with inter-laboratory reproducibility while maintaining clinical utility. Following CLSI guidelines, reported LoDs exhibit variability attributable to multiple methodological factors, including: (i) qPCR target selection (satDNA versus kDNA), (ii) *T. cruzi* strain or DTU used for sample spiking (e.g., TcI, TcII, TcVI), (iii) blood processing methods and reagents (anticoagulants/stabilizers such as EDTA or guanidine-containing buffers, boiled versus nonboiled sample treatment), and (iv) DNA extraction platform utilized (e.g., Roche, Qiagen, MagMax).

S1 Table summarizes LoD_95_ values from different laboratories using varied protocols, demonstrating the range of achievable analytical sensitivity and the factors contributing to variability.

LoD_95_ thresholds are based primarily on validation studies using reference strains, which are the most common DTUs in clinical studies from South America. Universal application of parasitic load determination across all DTUs (TcI through TcVI and TcBat) without correction may result in systematic under- or over-estimation of parasite loads depending on the DTU’s actual satDNA copy number. The implementation of a synthetic satDNA standard, such as the one developed by Muñoz-Calderón et al. [28] is a promising solution that was specifically designed to address DTU-independent quantification. This synthetic standard provides a uniform reference point that is not biased toward any particular DTU’s copy number variation, potentially enabling more consistent quantification across different parasite populations.

Quantification requires standardized calibration curves; optimal practice involves stable international reference materials.

### Quality assurance

qPCR laboratories must implement validated and periodically re-validated quality assurance procedures, including instrument calibration, reagent lot verification, use of positive and negative controls, technical replicates, and full documentation and traceability.

Distinct control functions are essential for quantitative comparability: (a) internationally accepted internal reference standards to be included in each qPCR assay run to control for inter-laboratory operational variability, instrument performance differences, and run-to-run variation, and (b) endogenous patient control genes (e.g., human housekeeping genes) to control for multiple quality control functions: detection of PCR inhibition, confirmation of adequate sample collection and DNA input, assessment of DNA extraction efficiency, and normalization for patient-to-patient variability in sample quality (Table 6).

Each laboratory should include reference standards at defined parasite load levels in every qPCR run to enable normalization and cross-laboratory comparability.

Moreover, endogenous controls can be used, beyond their current framing as inhibition detectors. This multi-functional quality control framework is essential for reliable quantification and result interpretation.

### Clinical sensitivity and specificity

In acute, congenital, and reactivation cases, qPCR sensitivity of ~98% is acceptable. In chronic CD, sensitivity of 60–70% compared with serology has been accepted, although >75% is the optimal target. Optimal thresholds should be targeted for: (i) registration trials intended for regulatory approval, where maximizing detection of treatment failures is critical for safety and efficacy determination; (ii) late-phase confirmatory studies; (iii) post-market surveillance programs; and (iv) clinical settings where treatment decisions depend heavily on qPCR results. Acceptable thresholds may be appropriate for: (i) early-phase exploratory trials (Phase I/II) where the primary objective is preliminary efficacy signal detection rather than definitive demonstration; (ii) proof-of-concept studies; (iii) situations where qPCR is used as one component of a composite endpoint rather than the sole outcome measure; and (iv) resource-limited settings where achieving optimal performance may not be feasible, provided that the limitations are explicitly acknowledged in result interpretation. It must be pointed out that these are general guidelines and that the appropriate threshold should be determined based on the specific study objectives, regulatory requirements, patient population characteristics, and risk-benefit considerations. Transparency in reporting actual achieved sensitivity (with confidence intervals) in trial publications is essential for proper interpretation of results and for meta-analyses combining data across studies. This is also relevant for sample size calculations that can incorporate realistic assumptions about qPCR sensitivity based on the specific patient population (acute versus chronic infection, disease stage, baseline parasitemia levels) and that sensitivity analyses should explore the impact of varying detection rates on statistical power.

Clinical sensitivity can be improved by serial sampling and replicate testing as strategies to improve detection sensitivity. Clinical specificity should remain ≥98%.

### Data interpretation and reporting

Standardized SOPs are required to harmonize data interpretation across trials, including Ct cutoffs, fluorescence thresholds, and quantification methods. Acceptable reporting times range from 48 hours to 1 week, with an optimal target of one working day.

### Genotyping

DTU genotyping should be regarded as a complementary analysis, particularly because it is difficult to resolve in blood samples from chronic CD patients, in whom parasitemia is generally below 5 par. eq./mL. This limitation reflects the fact that the molecular targets currently used to discriminate among DTUs are represented at substantially lower copy numbers than the targets used for *T. cruzi* detection.

## Discussion

Current trypanocidal therapies for CD remain suboptimal, reinforcing the need for safer and more effective regimens. Reliable tools to assess therapeutic efficacy are therefore essential. To date, DNA amplification methods—and qPCR in particular—are the accepted surrogate markers of treatment failure and have been widely used in Phase II and III trials [4–8,10,12–14]

For each trial, investigators should consider: (i) the analytical performance of their specific qPCR assay, (ii) the expected kinetics of parasitemia recurrence based on available data, (iii) practical feasibility and patient adherence to the proposed sampling schedules, and (iv) cost-effectiveness of different sampling strategies. Despite its widespread use, qPCR has not yet been universally accepted by regulatory agencies as a definitive registration endpoint. Beyond technical variability, international harmonization is challenged by the complex biology of *T. cruzi*, including its genetic diversity and structure, tissue tropism, and heterogeneous transmission patterns [29].

Persistence of infection is widely accepted as a driver of disease progression [30]. Given that serological cure may take decades, two complementary strategies are needed: strengthening and standardizing qPCR as a surrogate endpoint, and intensifying research into novel biomarkers of cure and disease progression. This consensus directly addresses the first objective.

Standardized qPCR implementation will enable future meta-analyses and improve comparability across trials. Recent modeling approaches based on serial qPCR data, including steady-state estimation and Bayesian hierarchical models, offer promising frameworks for evaluating treatment efficacy [31,32]

Technological advances, including commercial kits and automated extraction platforms, have improved reproducibility but have not eliminated heterogeneity. Adoption of WHO laboratory assessment tools and the revised MIQE guidelines [33], together with consensus-based performance criteria [34], can substantially strengthen data reliability. However, a critical field-wide gap remains: the absence of a WHO international standard for *T. cruzi* DNA, which limits inter-laboratory harmonization and regulatory standardization. Developing and internationally validating such reference material should be a priority. The synthetic satellite DNA (satDNA) standard for DTU-independent quantification is an important interim advance [28], but it has not yet been endorsed as a WHO international reference material. We propose this approach as a viable pathway toward the formal standard currently lacking in the field.

### Biological Constraints on Blood-Based Parasitological Response Endpoints in *T. cruzi* Infection

A sustained parasitological response paradigm is difficult to translate to *T. cruzi* infection because of parasite dormancy and tissue reservoirs in multiple compartments, including heart, skeletal muscle, adipose tissue, and gastrointestinal tract. Reactivation and replication are not synchronized across reservoirs, making parasite activity within a patient spatially and temporally heterogeneous. Therefore, clearance from peripheral blood does not ensure clearance from tissues and challenges the concept of declaring systemic “cure” using blood-based measurements alone.

A second limitation is that peripheral blood qPCR cannot detect or quantify tissue persistence. Parasites may persist with intermittent or subclinical parasitemia that escapes sampling, or remain confined to inaccessible reservoirs. Thus, a negative blood qPCR cannot be interpreted as evidence of systemic cure or sterilizing clearance.

Third, the relationship between blood-detectable parasites and total tissue parasite burden is uncertain and likely varies among individuals. Host immunity, tissue perfusion, parasite tropism, and treatment history may all influence this relationship, limiting the validity of blood-based surrogates as universal cure indicators.

Finally, these constraints make it difficult to distinguish sterilizing cure from temporary suppression of blood-detectable parasitemia. True sterilizing cure would require elimination of parasites from all reservoirs, which cannot currently be verified with blood-based diagnostics alone. Post-treatment blood negativity may therefore reflect suppression rather than eradication, with relapse possible after reservoir reactivation.

The implications for trial endpoints are clear: endpoints should be framed around early failure detection and relapse risk, not cure confirmation. True cure endpoints will require tissue-based diagnostics or multi-compartment biomarkers that better reflect total body parasite burden. Accordingly, the performance thresholds in our TPP should be interpreted as targets for detecting failure, not definitive cure criteria. Future work should prioritize multi-marker approaches, harmonized sampling where feasible, longer-term or nested analyses, tissue-based or multi-compartment biomarkers, imaging modalities, and longitudinal designs able to distinguish transient suppression from sustained clearance.

### Issues related to parasite genetic diversity and qPCR quantification

Most qPCR assays used in CD clinical trials target *T. cruzi* satDNA. However, satDNA copy number varies substantially across DTUs, which may affect parasite load quantification when parasite equivalents are estimated by assuming a fixed target copy number. Consequently, the LoD95 and quantification thresholds presented in Table 6 should not be interpreted as uniformly applicable across all DTUs.

In principle, DTU-specific correction factors could improve the accuracy of parasite load estimates when the infecting DTU is known or can be reliably determined. However, DTU genotyping is not routinely performed in most clinical or trial settings and is technically difficult to resolve in blood samples from chronic CD patients. Thus, although DTU-specific correction factors may be analytically relevant, their routine implementation is currently impractical. Accordingly, the thresholds proposed in Table 6 should be regarded as functional performance targets derived from the DTUs represented in validation studies, while recognizing that analytical sensitivity and quantification accuracy may vary according to regional DTU distribution [35]. For registration trials and regulatory submissions, this source of variability should be addressed by using DTU-independent reference standards, such as synthetic satDNA controls, characterizing DTUs in a representative subset of samples whenever feasible, and explicitly acknowledging parasite genetic diversity as a source of measurement uncertainty in the interpretation of qPCR results.

### Cost considerations

For CD clinical trials conducted in endemic countries, acceptable cost thresholds should prioritize accessibility, feasibility, and long-term sustainability over purely technical optimality, provided that the assay meets predefined and acceptable performance criteria. In this context, the most technically advanced option may not always be the most appropriate if its cost, infrastructure requirements, or supply-chain demands limit implementation in the settings where trials are conducted.

We do not propose specific prices as acceptable or optimal, given the marked heterogeneity in the economic conditions of affected countries and health systems. Actual costs may vary substantially according to geographic region, procurement volume, local taxation and import policies, shipping and customs procedures, availability of laboratory infrastructure, and institutional or public–private agreements. Therefore, a single universal cost threshold would be unlikely to reflect real-world implementation conditions across endemic settings.

Instead, we recommend that trial sponsors and implementing partners conduct region-specific cost assessments during trial planning. These assessments should consider not only reagent and consumable costs, but also equipment, maintenance, quality assurance, personnel training, sample transport, data management, and external quality assessment. Such an approach would help ensure that molecular monitoring strategies are both analytically reliable and operationally sustainable in the diverse contexts in which CD clinical trials are performed.

### Harmonization strategies for multi-center implementation

Thermocycler-related Ct variability limits the use of universal Ct cutoffs and complicates cross-trial comparability. Therefore, harmonization should rely on biological calibration rather than fixed Ct values. Each laboratory should validate instrument-specific Ct thresholds using common reference materials with defined parasite loads. These thresholds should then be used to translate platform-specific Ct values into standardized parasite-equivalent concentrations through calibration curves included in each qPCR run.

Before trial initiation, and periodically during the study, participating laboratories should complete inter-laboratory proficiency testing using identical blinded panels. Concordance should be assessed against predefined criteria, such as acceptable quantitative variation and agreement in positive/negative classification. Additional controllable variables should also be standardized across sites, including the use of a same SOP, same DNA extraction and amplification kits and quality control criteria.

For multi-center trials, centralized re-testing of a representative sample subset could provide an external quality check and identify systematic site- or instrument-related bias. Statistical analysis plans should prospectively assess inter-site variability and adjust for laboratory or equipment effects when needed. Trial reports should disclose thermocycler models, validation-derived Ct thresholds, quantitative results in parasite equivalents rather than raw Ct values, and proficiency testing concordance metrics. Overall, harmonization should prioritize standardized biological performance measures over instrument-dependent Ct values, while acknowledging residual equipment-related variability through predefined sensitivity analyses.

Finally, integrating qPCR endpoints with serological and emerging biomarkers will provide the most robust evidence of efficacy. In the interim, harmonized qPCR standards represent the most immediate path forward. Establishing a TPP for qPCR in CD will reduce variability, facilitate regulatory alignment, and accelerate development of urgently needed therapies.

## Supporting information

S1 Table Analytical sensitivity of qPCR assays for *T. cruzi* DNA detection in blood samples. LoD_95_ values have been estimated by probit or logistic regression, as reported in each source. SatDNA, satellite DNA; kDNA, minicircle DNA.
(DOCX)
