# Supplementary material for: Consensus framework for developing a target product profile of real-time PCR in Chagas disease therapeutic monitoring
Source: PLoS Negl Trop Dis. 2026 Jul 9;20(7):e0014452. doi: 10.1371/journal.pntd.0014452 (PMC13349099; doi:10.1371/journal.pntd.0014452)
Supplement: S1 Table — LoD95 values have been estimated by probit or logistic regression, as reported in each source. SatDNA, satellite DNA; kDNA, minicircle DNA. (DOCX) [file pntd.0014452.s001.docx]

**S1 Table. Analytical sensitivity of qPCR assays for *T. cruzi* DNA detection in blood samples.**

| **Blood sample condition** | **DNA extraction method** | **PCR mixture** | **qPCR target** | **LoD_95_** | **Strain (DTU)** | **Reference** |
| --- | --- | --- | --- | --- | --- | --- |
| Guanidine-EDTA blood (GEB), boiled | High Pure PCR Template Preparation Kit (Roche Diagnostics) | In-house qPCR | kDNA | 0.16 par. eq./mL | CL-Brener (TcVI) | [19] |
| Guanidine-EDTA blood (GEB), non-boiled | High Pure PCR Template Preparation Kit (Roche Diagnostics) | In-house qPCR | kDNA | 0.23 par. eq./mL | CL-Brener (TcVI) | [19] |
| Guanidine-EDTA blood (GEB), boiled | High Pure PCR Template Preparation Kit (Roche Diagnostics) | In-house qPCR | SatDNA | 0.46 par. eq./mL | CL-Brener (TcVI) | [18, 19] |
| Guanidine-EDTA blood (GEB), non-boiled | High Pure PCR Template Preparation Kit (Roche Diagnostics) | In-house qPCR | SatDNA | 0.69 par. eq./mL | CL-Brener (TcVI) | [18, 19] |
| Guanidine-EDTA blood (GEB), boiled | High Pure PCR Template Preparation Kit (Roche Diagnostics) | BioMol Chagas kit, IBMP, Brazil | SatDNA | 0.16 par. eq./mL | Y (Tc II) | [25] |
| Guanidine-EDTA blood (GEB), boiled | High Pure PCR Template Preparation Kit (Roche Diagnostics) | In-house qPCR | SatDNA | 0.64 par. eq./mL | Y (Tc II) | [25] |

LoD_95_ values have been estimated by Probit regression analysis, as reported in each source. SatDNA:satellite DNA; kDNA: minicircle DNA; Guanidine-EDTA:

Guanidine hydrochloride 6 M, EDTA 0.2 M, pH: 8.00; DTU: Discrete Typing Unit.
